# Metformin selectively dampens the acute inflammatory response through an AMPK-dependent mechanism

**DOI:** 10.1038/s41598-021-97441-x

**Published:** 2021-09-21

**Authors:** Thomas S. Postler, Vincent Peng, Dev M. Bhatt, Sankar Ghosh

**Affiliations:** 1 Department of Microbiology & Immunology, Vagelos College of Physicians & Surgeons, Columbia University Irving Medical Center, New York, NY 10032 USA; 2 Present Address: Department of Pathology and Immunology, Washington University School of Medicine, St. Louis, MO 63110 USA; 3 Present Address: Amgen Research Oncology and Inflammation, South San Francisco, CA 94080 USA

**Keywords:** Endocrine system and metabolic diseases, Immunological disorders, Metabolic disorders

## Abstract

Metformin is a first-line drug in the treatment of type-2 diabetes mellitus (T2DM). In addition to its antigluconeogenic and insulin-sensitizing properties, metformin has emerged as a potent inhibitor of the chronic inflammatory response of macrophages. In particular, metformin treatment has been shown to reduce expression of interleukin (IL-) 1β during long-term exposure to the pro-inflammatory stimulus lipopolysaccharide (LPS) through a reduction in reactive oxygen species (ROS), which decreases the levels of the hypoxia-inducible factor (HIF) 1-α, and through enhanced expression of IL-10. However, the effect of metformin on the acute inflammatory response, before significant levels of ROS accumulate in the cell, has not been explored. Here, we show that metformin alters the acute inflammatory response through its activation of AMP-activated protein kinase (AMPK), but independently of HIF1-α and IL-10, in primary macrophages and two macrophage-like cell lines. Thus, metformin changes the acute and the chronic inflammatory response through fundamentally distinct mechanisms. Furthermore, RNA-seq analysis reveals that metformin pretreatment affects the levels of a large yet selective subset of inflammatory genes, dampening the response to short-term LPS exposure and affecting a wide range of pathways and biological functions. Taken together, these findings reveal an unexpected complexity in the anti-inflammatory properties of this widely used drug.

## Introduction

The global prevalence of diabetes has nearly doubled since 1980, reaching 8.5% in 2014. The majority of these 422 million patients suffer from type-2 diabetes mellitus (T2DM), which is the primary driver of the continuing increase in diabetes prevalence^1^. T2DM is characterized by hyperglycemia resulting from progressive insulin resistance and dysfunction of pancreatic β cells. Additionally, T2DM is associated with low-level, systemic inflammation, and these inflammatory processes contribute to T2DM pathogenesis^2,3^. In particular, the cytokine interleukin (IL)-1β has been shown to be an important contributor to the inflammatory component of T2DM^4,5^. Specifically, IL-1β directly reduces the sensitivity of adipocytes to insulin and induces the death of pancreatic β cells^6–10^. Consistent with these observations, inhibition of IL-1β signaling has exhibited protective effects in mouse models of T2DM and in patients^11–13^. Multiple cell types are believed to contribute to the release of IL-1β and other pro-inflammatory cytokines in T2DM patients, most notably macrophages associated with white adipose tissue^14,15^.

Secretion of IL-1β is tightly regulated by two separate mechanisms: transcription (priming) and proteolytic processing of a precursor protein by the inflammasome^16^. Pro-inflammatory stimuli chronically present in the tissue of T2DM patients, such as free fatty acids and the bacterial cell-wall component lipopolysaccharide (LPS), induce transcription of *Il1b* and other cytokine-encoding genes^17,18^. This results in synthesis of the inactive form pro-IL-1β, which must then be cleaved by caspase-1 after inflammasome activation to yield the active, secreted IL-1β molecule^16^. The NLRP3 inflammasome in particular has been shown to be activated by a variety of stimuli present during T2DM, including islet amyloid peptide, fatty acids and ceramide^19–23^. Previous studies exploring the relationship between LPS and IL-1β expression in macrophages found that prolonged LPS exposure results in an accumulation of succinate in mitochondria and elevated mitochondrial membrane potential, which together drive increased production of reactive oxygen species (ROS). This increase in ROS stabilizes hypoxia-inducible factor (HIF) 1-α, which directly enhances transcription of *Il1b*^24–26^.

Decades after its use became widespread in the 1990s, metformin is still the typical first choice for initial treatment of newly diagnosed T2DM and is commonly included in subsequent long-term combination regimens as well. This success is based on metformin’s efficacy at lowering blood glucose, but also on its favorable safety profile and comparatively low cost^27,28^. Primarily, metformin treatment inhibits hepatic gluconeogenesis and sensitizes peripheral tissues to insulin. Furthermore, it has been reported to contribute to long-term weight loss and may exhibit modest cardioprotective effects, along with a plethora of other advantageous properties^27,29–40^.

Despite widespread use and significant research interest, the molecular mechanisms by which metformin mediates all these effects remain incompletely understood^27^. However, one well-established mode of metformin action is the inhibition of NADH:ubiquinone oxidoreductase, also known as mitochondrial complex I of the respiratory chain^41–43^. This inhibition results in two major, independent metabolic changes: (i) a decrease in ATP production, leading to increased intracellular AMP levels and thus activation of AMP-activated protein kinase (AMPK); and (ii) a selective decrease in reverse electron flux at complex I resulting in reduced mitochondrial ROS production^24,39,44–47^. Intriguingly, this second property has been shown to reduce *Il1b* transcript levels during chronic exposure to LPS, opening up the possibility that the systemic benefits of metformin treatment may be enhanced by its anti-inflammatory properties^24^.

While the effects of metformin on the later stages of the inflammatory response are well characterized, relatively little is known about whether and how the drug modulates the early phase, before significant levels of ROS are generated. We therefore endeavored to investigate whether metformin alters *Il1b* transcript levels during the acute LPS response in macrophages. Metformin pretreatment did indeed reduce *Il1b* at only 2 h of LPS stimulation. However, unlike what has been observed during chronic LPS stimulation, this effect was mediated by AMPK activation and did not require the upregulation of *Il10* or stabilization of HIF1-α. Beyond *Il1b*, metformin had a profound effect on the expression of a large but selective subset of inflammatory genes. Among the metformin-sensitive genes, expression of those upregulated by LPS was mostly inhibited by metformin, while expression of those downregulated by LPS was mostly enhanced, thus dampening the acute LPS response overall. The anti-inflammatory properties of metformin are therefore more complex than previously thought, and mediated by distinct mechanisms during the acute and the chronic LPS response.

## Results

### During the acute LPS response, metformin reduces transcript levels of *Il1b* independently of IL-10, HIF1-α, and NF-κB

**Figure 1:**
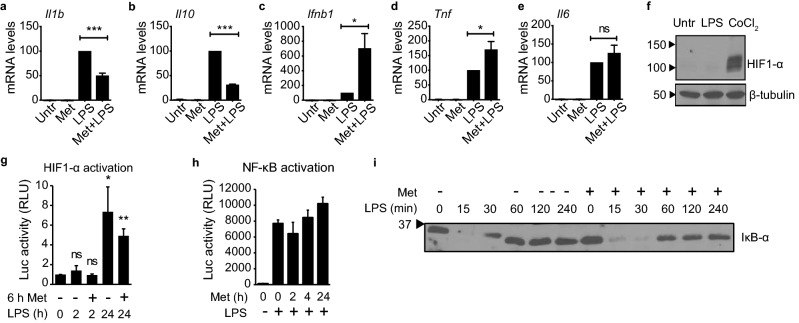
Metformin alters *Il1b* transcript levels during the acute LPS response independently of IL-10, HIF1-α, and NF-κB. (**a–e**) RT-qPCR with RNA from primary BMDMs, quantifying the indicated genes. Cells were pretreated with 3 mM metformin for 6 h, followed by stimulation with 100 ng/ml LPS for 2 h (Met + LPS). Controls were either left untreated (Untr), treated with metformin but not stimulated (Met), or stimulated with 100 ng/ml LPS for 2 h in the absence of metformin pretreatment (LPS). *Tubb5* served as reference gene. Error bars indicate standard error of 5 independent experiments. (**f**) Western blot of lysates from J774 cells treated with 100 ng/ml LPS for 2 h or with 100 μM CoCl_2_ for 4 h, probing for HIF1-α and β-tubulin. Black arrows indicate molecular weight in kDa. Images were cropped. See Supplementary Fig. S1f,g for full lanes. (**g,h**) Luciferase assay with RAW 264.7 cells (**g**) transiently transfected with the pGL2-HRE-Luc HIF1-α-dependent luciferase reporter construct or (**h**) stably transfected with the pBIIx-Luc NF-κB-dependent luciferase reporter construct. Cells were (**g**) pretreated or not with 5 mM metformin (Met) for 6 h and then stimulated with 100 ng/ml LPS for the indicated times up to 24 h, or (**h**) pretreated with 1 mM metformin (Met) for the indicated times up to 24 h and then stimulated or not with 100 ng/ml LPS for 2 h. Luc, luciferase; RLU, relative luminescence units. Error bars indicate standard deviation of (**g**) 5 or (**h**) 3 independent experiments. (**i**) Western blot analysis of IκB-α in lysates of J774 macrophages. Cells were treated with 10 mM metformin for 30 min and stimulated with 100 ng/ml LPS for up to 4 h. Protein concentrations in lysates were quantified and equivalent amounts loaded in each lane. Black arrow indicates molecular weight in kDa. Image was cropped. See Supplementary Fig. S1h for uncropped lanes. p-values: ***p < 0.001; **p < 0.01; *p < 0.05; *ns* not significant.

To ascertain whether metformin affects the acute inflammatory response, we began by determining the transcript levels of *Il1b* in primary bone-marrow-derived macrophages (BMDMs). Cells were pretreated with metformin for 6 h, to mimic the long-term treatment typical for T2DM patients, and then exposed to LPS for 2 h. Indeed, metformin pretreatment significantly reduced *Il1b* levels even with such short LPS stimulation (Fig. 1a). This effect was dose-dependent across a range of concentrations typically used to interrogate metformin in cell culture (Supplementary Fig. S1a)^24,39,48^. Metformin has been reported to reduce transcript levels of *Il1b* during chronic LPS stimulation as well, an effect that was found to depend on the metformin-induced increase in *Il10* transcript levels observed at later time points^24^. Surprisingly, however, *Il10* was downregulated during short-term LPS stimulation subsequent to metformin pretreatment (Fig. 1b). This first implied that metformin might reduce the transcript levels of *Il1b* during the acute LPS response through a different mechanism than during chronic LPS exposure. Metformin did not inhibit the LPS response globally, as *Ifnb1* and *Tnf* were upregulated, while *Il6* remained unchanged (Fig. 1c–e). These observations were recapitulated in the macrophage-like cell line J774 with the exception of *Il6*, which exhibited a modest but statistically significant increase of transcript levels in these cells (Supplementary Fig. S1b–e). Thus, metformin reduces *Il1b* transcript levels during the acute LPS response independently of an increase in *Il10* transcript levels.

Next, we investigated the role of HIF1-α in this phenomenon. HIF1-α has been shown to bind directly to the *Il1b* promoter and enhance transcription in macrophages during chronic LPS exposure^25,26^. This is mediated by LPS-induced production of ROS, which stabilizes HIF1-α protein levels. As metformin inhibits ROS production, metformin might affect *Il1b* transcript levels by preventing ROS-mediated HIF1-α stabilization^24^. Indeed, a recent study found that metformin treatment results in a reduction of HIF1-α protein in human gall-bladder cancer GBC-SD cells^49^. We therefore tested whether short-term LPS exposure was sufficient to stabilize HIF1-α. After 2 h of LPS stimulation, no change in HIF1-α levels was detectable compared to untreated cells, while treatment with the HIF1-α inducer CoCl_2_ resulted in a strong increase (Fig. 1f, Supplementary Fig. S1f,g). To measure HIF1-α-dependent transcription more directly, we transiently transfected macrophage-like RAW 264.7 cells with a luciferase expression construct under control of the Hypoxia Response Element (HRE). Stimulation with LPS for 24 h resulted in robust HIF1-α-driven luciferase activity in this system, which was markedly reduced in cells pretreated with metformin. Exposure to LPS for 2 h, however, did not increase luciferase levels significantly, further corroborating that HIF1-α does not contribute meaningfully to the acute LPS response (Fig. 1g). This is consistent with the observation by Tannahill et al*.* that HIF1-α does not bind to the promoter of *Il1b* after 2 h of LPS stimulation, but does so only after 4 h and later^26^. These results indicate that the effect of metformin on transcript levels during the acute LPS response is not mediated by HIF1-α, but instead through a mechanism that is distinct from what has been reported for chronic LPS exposure.

The NF-κB family of transcription factors comprises central regulators of the inflammatory response^50–52^. Accordingly, many LPS-responsive genes are known NF-κB target genes, including *Il1b*, *Il10*, *Ifnb1*, *Tnf*, and *Il6*^53–60^. As some of these NF-κB-dependent genes were downregulated by metformin pretreatment while others were upregulated or unaffected, NF-κB activation itself is unlikely to be altered by metformin (Fig. 1a-e and Supplementary Fig. S1b–e). To test this prediction, we pretreated RAW 264.7-derived NF-κB reporter cells with metformin for up to 24 h and determined the level of NF-κB activation after 2 h of LPS stimulation. Metformin pretreatment did not affect NF-κB activation at any of the examined time points (Fig. 1h). We further assessed the effect of metformin pretreatment on the protein levels of IκB-α, a primary inhibitor of NF-κB activation whose expression is directly controlled by NF-κB. LPS stimulation is known to result in rapid degradation of IκB-α, followed by an accumulation of newly synthesized protein to form a negative feedback loop^61–68^. Metformin pretreatment did not significantly alter the degradation kinetics of IκB-α during short-term LPS exposure up to 4 h, confirming that the signaling cascade resulting in NF-κB activation was unaffected (Fig. 1i, Supplementary Fig. S1h). Only a slight decrease in the amount of newly synthesized IκB-α protein was noted at later time points. These minor effects cannot account for the more dramatic and antipodal changes in the transcript levels of the examined NF-κB-dependent genes. Consequently, these results establish that metformin does not act by directly blocking NF-κB activation.

### Metformin alters transcript levels during the acute LPS response by activating AMPK

**Figure 2:**
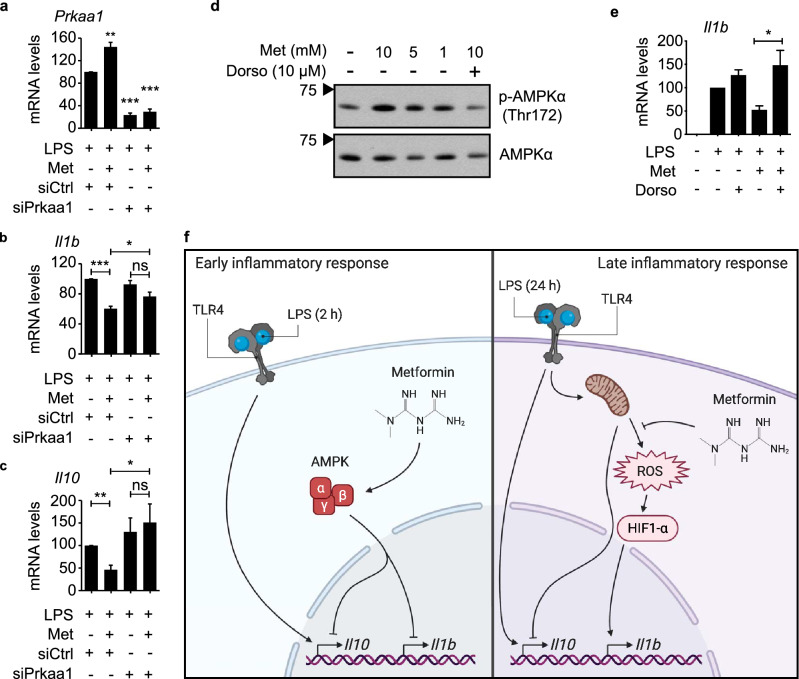
Metformin alters transcript levels during the acute LPS response by activating AMPK. (**a–c**) RT-qPCR with RNA from RAW 264.7 cells, quantifying expression of the indicated genes. Cells were transfected with siRNA targeting *Prkaa1* (siPrkaa1) or a control siRNA (siCtrl), pretreated or not with 5 mM metformin (Met) for 6 h, and stimulated with 100 ng/ml LPS for 2 h. *Rplp0* served as reference gene. (**d**) Western blot of lysates from primary BMDMs treated with metformin (Met) and dorsomorphin (Dorso) for 6 h, probing for phospho-AMPKα (Thr172) and total AMPKα. Black arrows indicate molecular weight in kDa. Images were cropped. See Supplementary Fig. S1j,k for full lanes. (**e**) RT-qPCR with RNA from J774 cells, quantifying expression of *Il1b*. Cells were pretreated with 5 mM metformin (Met) and/or 10 μM dorsomorphin (Dorso) for 6 h, followed by stimulation with 100 ng/ml LPS for 2 h. *Tubb5* served as reference gene. (**f**) Model of the mechanism through which metformin affects the inflammatory response of macrophages. During the early phase, metformin reduces the transcription of *Il1b* and *Il10* by activating AMPK (left panel). During the late phase, metformin reduces the production of ROS by mitochondria, which limits protein levels of HIF1-α and results in decreased expression of *Il1b*, whereas expression of *Il10* is enhanced (right panel). p-values: ***p < 0.001; **p < 0.01; *p < 0.05; *ns* not significant. Error bars indicate standard error of 5 independent experiments.

As no changes in the protein level or transcriptional activity of HIF1-α could be detected after 2 h of LPS stimulation, it is unlikely that HIF1-α mediates the effect of metformin on *Il1b* transcript levels during the acute LPS response. We therefore examined whether AMPK activation, the other main effect of metformin treatment, might be responsible for the observed phenotype. We used siRNA to knock down expression of *Prkaa1*, the gene encoding the only catalytic AMPK subunit expressed in macrophages (Fig. 2a, Supplementary Fig. S1i)^69–72^. Strikingly, this reduction of AMPKα levels was sufficient to counteract the effect of metformin pretreatment on *Il1b* and *Il10* expression during short-term LPS stimulation of RAW 264.7 cells (Fig. 2b,c). To validate the involvement of AMPK further, we treated cells simultaneously with metformin and the selective AMPK inhibitor dorsomorphin, also known as compound C. As expected, treatment with metformin led to a dose-dependent increase in AMPKα phosphorylation at Thr172, a marker of AMPK activation, which was abrogated by dorsomorphin (Fig. 2d, Supplementary Fig. S1j,k). Similar to the knockdown of *Prkaa1*, dorsomorphin reversed the reduction of *Il1b* transcript levels by metformin (Fig. 2e). Conversely, transcript levels of the metformin-upregulated genes *Ifnb1* and *Tnf* were significantly decreased (Supplementary Fig. S1l,m). These data demonstrate that metformin alters the acute LPS response at least partly through activation of AMPK. As AMPK has been shown to be fully dispensable for metformin-mediated downregulation of *Il1b* during chronic LPS exposure, this finding further corroborates that metformin alters the acute and the chronic LPS response through distinct mechanisms (Fig. 2f)^24^.

### Metformin dampens the acute LPS response broadly yet selectively

Next, we used RNA-seq analysis to assess the global effect of metformin on the acute LPS response in macrophages (Fig. 3, Supplementary Fig. S2). To establish the baseline response to LPS, we first compared the transcriptome of untreated primary BMDMs with the transcriptome of BMDMs stimulated with LPS for 2 h. LPS treatment altered the expression levels of 1390 genes by at least fourfold with an adjusted p-value of less than 0.01 (Fig. 3a–c, Supplementary Table S1). This stringent fold-change cut-off ensured that only highly LPS-responsive genes would be considered in the downstream analysis. The majority of these genes (1003) exhibited increased expression; the remainder were downregulated by LPS stimulation. Metformin pretreatment altered the expression of 1251 genes overall by at least 1.6-fold with an adjusted p-value of less than 0.01, compared to BMDMs treated only with LPS (Fig. 3c–e, Supplementary Table S2). We chose this less conservative fold-change cut-off as metformin was expected to alter gene expression more subtly than the fundamental transcriptional reorganization elicited by LPS stimulation. Intriguingly, approximately a quarter of the metformin-sensitive genes (314) overlapped with the 1390 LPS-responsive genes (Fig. 3c,e, Supplementary Table S3). Considering that metformin is primarily known for its effects on metabolic pathways, this proportion was unexpectedly high. These numbers are also surprising because they demonstrate that nearly 23% of LPS-responsive genes are sensitive to metformin pretreatment, indicating a substantial impact on the inflammatory response beyond individual genes. The effect of metformin largely antagonized LPS stimulation: transcript levels of 178 out of 212 of metformin-sensitive, LPS-upregulated genes were reduced by metformin pretreatment, whereas transcript levels of 99 out of 102 of metformin-sensitive, LPS-downregulated genes were increased by metformin pretreatment (Fig. 3b,c, Supplementary Fig. S2b). There was only a modest negative correlation between the magnitude of metformin sensitivity and the magnitude of LPS responsiveness, indicating that metformin does not simply affect genes that respond to LPS particularly well or particularly poorly (Supplementary Fig. S2b). Thus, metformin exerts a broad yet selective dampening effect on the acute LPS response.

**﻿Figure 3:**
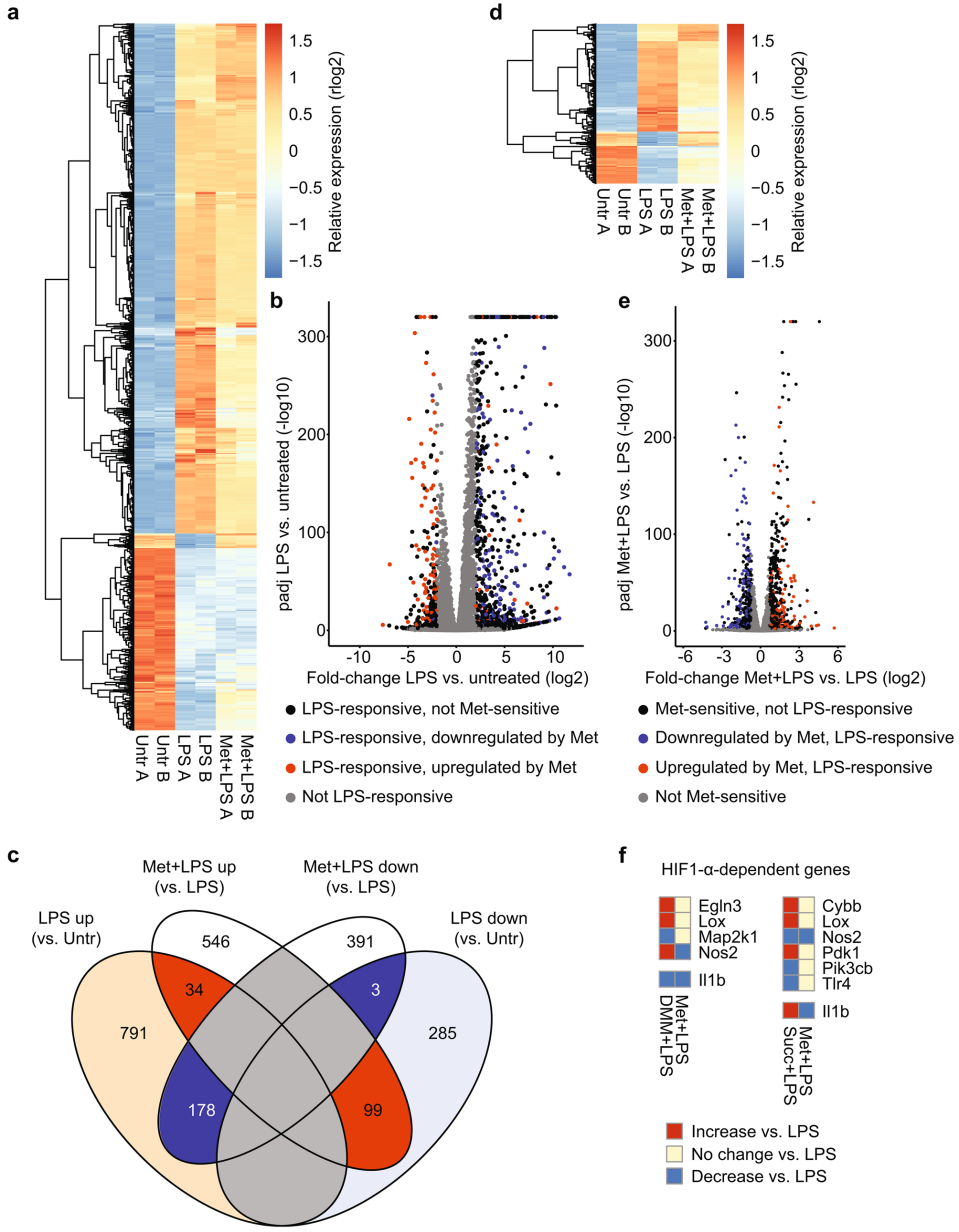
RNA-seq reveals an unexpectedly broad impact of metformin on the acute LPS response. Primary BMDMs were pretreated with 5 mM metformin for 6 h, followed by stimulation with 100 ng/ml LPS for 2 h (Met + LPS). Controls were either left untreated (Untr) or stimulated with 100 ng/ml LPS for 2 h in the absence of metformin pretreatment (LPS). RNA-seq was performed in duplicate for each group. (**a**) Expression heatmap with hierarchical clustering of all LPS-responsive genes. Colors represent regularized, log2-transformed counts (rlog2) after normalization per row. (**b**) Volcano plot of log2-transformed fold-change between LPS-treated BMDMs vs. untreated BMDMs and corresponding adjusted p-values (padj). padj values < 10^–320^ were set to equal 10^–320^. Note negative logarithmic scale on y-axis. Colors indicate LPS responsiveness and metformin sensitivity, as specified. (**c**) Venn diagram of all LPS-responsive and all metformin-sensitive genes with direction of fold-change. (**d**) Expression heatmap with hierarchical clustering of all genes that were both LPS-responsive and metformin-sensitive. Colors represent rlog2 counts after normalization per row. (**e**) Volcano plot of log2-transformed fold-change between metformin- and LPS-treated BMDMs vs. LPS-treated BMDMs and corresponding padj values. padj values < 10^–320^ were set to equal 10^–320^. Note negative logarithmic scale on y-axis. Colors indicate LPS responsiveness and metformin sensitivity, as specified. (**f**) Comparison of the effect of metformin (Met), dimethyl malonate (DMM) and diethyl succinate (Succ) on the LPS response at 2 h (for Met; RNA-seq data from this study) and 48 h (for DMM and Succ; RNA-seq data published by Mills et al*.*^25^). Only genes with a log2-transformed fold-change > 0.7 in the data set of Mills et al*.* were included (regardless of the false-discovery rate). Colors indicate direction of change compared to samples treated only with LPS.

To interrogate further the conclusion that HIF1-α was not involved in the effect on transcript levels exhibited by metformin pretreatment during the acute LPS response, we examined HIF1-α-dependent genes in our RNA-seq data set. Mills et al*.* have demonstrated that enhancing ROS production during prolonged LPS exposure by pretreatment with diethyl succinate alters transcript levels of several HIF1-α target genes^25^. Blocking ROS production by pretreatment with dimethyl malonate generally has the opposite effect, although not necessarily to the same extent. We therefore compared the effect of metformin treatment prior to acute LPS exposure (our RNA-seq data set) to the effect of diethyl succinate and dimethyl malonate during chronic LPS exposure (Mills et al*.*) on transcript levels of HIF1-α-dependent genes^25^. If metformin exerted its effects through inhibition of ROS production and therefore HIF1-α, dimethyl malonate and metformin would be expected to have the same effect on transcript levels (as is the case for *Il1b*), whereas diethyl succinate should have an opposite phenotype. However, most genes altered by pretreatment with dimethyl malonate during chronic LPS stimulation were not affected by metformin pretreatment during acute LPS stimulation. The sole exception from this was *Nos2*, which was altered in opposite directions by metformin and dimethyl malonate. Similarly, genes whose transcript levels were affected by pretreatment with diethyl succinate were mostly unaffected by metformin pretreatment. The exception was again *Nos2*, which was downregulated after both diethyl succinate and metformin pretreatment (Fig. 3f). Thus, the results from this RNA-seq analysis corroborate the finding that HIF1-α does not mediate the effect of metformin pretreatment on the acute inflammatory response.

### Metformin pretreatment affects inflammatory genes involved in a wide range of pathways, biological functions and diseases

To assess the functional consequences of the transcriptional changes to the acute LPS response elicited by metformin treatment, we performed Ingenuity Pathway Analysis (IPA) to identify canonical pathways, biological functions and diseases involving LPS-responsive, metformin-sensitive genes^73^. As the LPS response predominantly increases transcriptional activity (Fig. 3b,c), we focused our attention on the LPS-responsive genes downregulated after metformin pretreatment. To distinguish between enrichment based on LPS responsiveness and metformin sensitivity, we used only the set of 1390 LPS-responsive genes as background, rather than the entirety of expressed genes.

**Figure 4:**
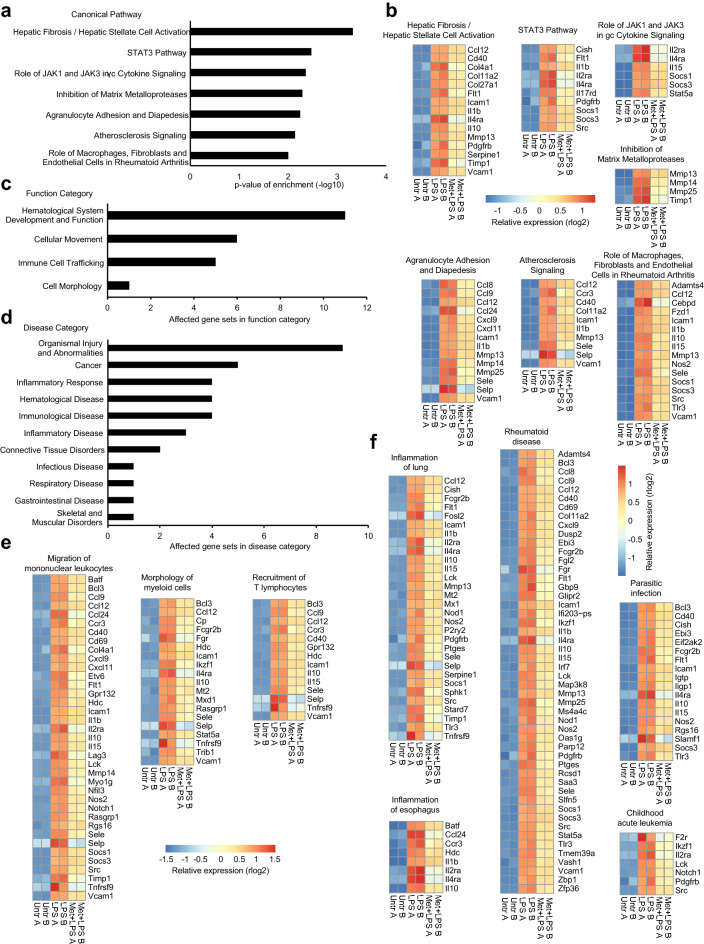
LPS-responsive genes downregulated by metformin are associated with a wide range of pathways, biological functions and diseases. Genes downregulated by metformin were investigated with Ingenuity Pathway Analysis. The set of 1390 LPS-responsive genes served as background against which enrichment was calculated. (**a,b**) Canonical Pathways enriched for metformin-downregulated genes. (**a**) List of affected pathways with the corresponding p-value of enrichment. (**b**) Alphabetical expression heatmaps of metformin-downregulated genes in the respective canonical pathway. Colors represent regularized, log2-transformed counts (rlog2) after normalization per row. (**c,d**) List of (**c**) function and (**d**) disease categories of gene sets enriched for metformin-downregulated genes. Shown is the number of gene sets belonging to each category (enrichment p-value < 10^–3^). Note that several gene sets belong into more than one category. See Supplementary Table S4 for a complete list of gene sets, associated categories and enrichment p-values. (**e,f**) Alphabetical expression heatmaps of metformin-downregulated genes in the respective gene sets. Shown are examples from the (**e**) function and the (**f**) disease categories. The remaining gene sets are shown in Supplementary Fig. S3b. Colors represent rlog2 counts after normalization per row.

IPA identified 7 canonical pathways enriched for genes downregulated by metformin; the number of metformin-sensitive genes in these pathways ranged from 4 to 16 (Fig. 4a,b). Notably, regulation of the transcription factor STAT3 was implicated by two distinct but overlapping gene sets: the STAT3 pathway itself and the pathway describing the role of JAK1 and JAK3 in γc cytokine signaling, which act as regulators of STAT proteins including STAT3. Unexpectedly, an effect of metformin on cellular migration was indicated by the enrichment of metformin-downregulated genes in the pathways describing inhibition of matrix metalloproteases (MMPs) and agranulocyte adhesion and diapedesis. This is also consistent with an effect on the canonical STAT3 pathway, as STAT3 has previously been implicated in the migration of macrophages^74,75^.

IPA for biological functions and diseases identified 29 gene sets enriched for metformin-downregulated genes (Supplementary Fig. S3a). These were partly overlapping, ranged in size from 6 to 60 genes and represented 4 broader functional categories and 11 disease categories (Fig. 4c,d, Supplementary Table S4). The gene sets in the functional categories indicated a substantial effect of metformin on cellular movement and immune-cell trafficking, as well as cell morphology (Fig. 4c,e, Supplementary Fig. S3b). For instance, 6 distinct but overlapping gene sets were associated with cellular movement, including a gene set involved in the migration of mononuclear leukocytes. Most of these gene sets were also associated with immune-cell trafficking.

The 11 categories of disease-associated gene sets included a broad range of inflammatory and (auto-)immune-related pathologies, including inflammation of lung and esophagus, rheumatoid disease, and parasitic infection. Several overlapping gene sets associated with cancer and hematological disease, all pertaining to leukemia, were also enriched for metformin-downregulated genes (Fig. 4d,f, Supplementary Fig. S3b). These results are not surprising, because all genes included in this analysis were LPS-responsive; but they underscore the far-reaching effects of the anti-inflammatory properties of metformin in vivo.

## Discussion

Hundreds of millions of patients worldwide suffer from T2DM, and the drug most commonly prescribed to treat them is metformin. Gaining comprehensive insight into metformin’s multiple mechanisms of action is therefore of considerable importance, as it may enable us to improve upon the efficacy of the drug and further expand our understanding of T2DM pathogenesis. To our knowledge, this is the first study specifically investigating the effect of metformin on the acute LPS response.

As has previously been shown for the chronic LPS response, *Il1b* transcript levels were reduced by metformin treatment preceding acute LPS stimulation of primary macrophages, as well as two macrophage-like cell lines^24^. The underlying mechanism, however, was fundamentally different. While the importance of IL-10 and ROS/HIF1-α has been established for the effect of metformin on *Il1b* transcript levels during chronic stimulation, involvement of neither could be detected in the metformin-mediated reduction of *Il1b* at 2 h of LPS exposure^24–26^. Instead, activation of AMPK, which is dispensable for the inhibition of *Il1b* by metformin during the chronic LPS response, was required for this effect at 2 h of LPS stimulation. Knockdown of *Prkaa1*, the only catalytic AMPK subunit expressed in macrophages, reversed the effect of metformin treatment on expression of both *Il1b* and *Il10*^69–72^. Similarly, blocking AMPK activation with dorsomorphin abrogated the effect of metformin on *Il1b*, as well as *Ifnb1* and *Tnf*. Kim et al*.* have previously reported similar results for *Tnf* and *Il6* with 4 h of LPS stimulation^48^. Dorsomorphin is considered a highly selective inhibitor of AMPK activation and is commonly used to antagonize AMPK activation by metformin^46^. Nonetheless, it should be noted that inhibitory effects on bone morphogenetic protein (BMP) signaling have also been reported, along with other AMPK-independent properties^76–80^.

There is no clear consensus on the physiological concentrations of metformin circulating in T2DM patients receiving the drug, and even less is known about the extent to which it accumulates in tissues^81–83^. Nonetheless, the metformin concentrations that are commonly used for in vitro studies, including the present work, likely are higher than the concentrations found in vivo^24,39,48,84^. However, it is important to note that metformin is a metabolic drug, and the level of sensitivity to its effects depends to a large extent on the metabolic state of the exposed cell^84,85^. Nutrients are available in excess to cells in vitro but not in vivo, which offers a compelling explanation for the discrepancy in the effective dose of metformin in patients and in cell culture.

On the transcriptome level, our results demonstrate that metformin exerts a broad yet selective effect on the acute LPS response, as the transcript levels of over 20% of LPS-responsive genes were significantly altered. Metformin largely antagonized the effects of LPS: the vast majority of metformin-sensitive genes exhibited expression changes in the opposite direction of the LPS response. As LPS is an extremely potent pro-inflammatory stimulus, this observation is consistent with previous reports of anti-inflammatory properties of metformin^24,39,48,86^. We extend these findings by showing the full breadth of metformin-sensitive genes during the acute LPS response. Intriguingly, several LPS-responsive anti-inflammatory genes were also downregulated by metformin pretreatment, most notably *Il10*, *Socs1* and *Socs3*. This reveals that the effects of metformin on the LPS response are multifaceted and more complex than previously thought.

The downregulation of LPS-induced genes by metformin was predicted to affect a range of canonical signaling pathways. The pathway with the highest enrichment of metformin-downregulated genes was associated with hepatic fibrosis or hepatic stellate cell activation. This may be of clinical interest, considering that T2DM patients are at higher risk of hepatic fibrosis^87^. The enrichment of metformin-downregulated genes in the STAT3 pathway and the pathway describing the role of JAK1 and JAK3 in γc cytokine signaling implicate STAT3 as a direct or indirect target of metformin action. This is consistent with previous reports that AMPK activation by metformin reduces phosphorylation of STAT3 Y705, and thus STAT3 activation, in other cell types^34,88,89^. Interestingly, STAT3 is involved in the expression of pro-inflammatory genes, but also acts as an important mediator of the anti-inflammatory properties of IL-10^90–92^. Additionally, STAT3 has been implicated in the migration of macrophages, which further supports the unexpected finding that metformin treatment affected a gene set associated with agranulocyte adhesion and diapedesis^74,75^. The enrichment of metformin-downregulated genes in the pathway characterizing the inhibition of MMPs is also in agreement with an effect on cellular migration. It is therefore not surprising that gene sets functionally associated with cellular movement and immune-cell trafficking were metformin-sensitive as well, including the gene sets involved in the migration of mononuclear leukocytes and the recruitment of T lymphocytes. This convergence of evidence that metformin may inhibit macrophage migration and lymphocyte recruitment presents a novel aspect of metformin action and warrants further studies.

As all the genes investigated with IPA had been pre-selected to be LPS-responsive, it was expected that inflammatory and (auto-)immune-related diseases would feature prominently among the results. Indeed, gene sets associated with inflammation of lung and esophagus, rheumatoid disease, parasitic infection and various forms of leukemia all were enriched for metformin-downregulated genes, as were the canonical pathways describing atherosclerosis signaling and the role of macrophages, fibroblasts and endothelial cells in rheumatoid arthritis. This reflects the impressive breadth of metformin’s potential effects on inflammatory processes in vivo.

Together, these findings reveal that the effect of metformin on the inflammatory response is more complex than previously thought and indeed relies on different mechanisms at different stages. Given the immense public-health relevance of metformin, comprehensively defining the distinct pathways through which this widely used drug exerts its physiological effects remains a priority.

## Methods

### Isolation of BMDMs from mice and cell culture

Wildtype C57BL/6 mice were purchased from The Jackson Laboratory and housed in a facility accredited by the Association for Assessment and Accreditation of Laboratory Animal Care International at Columbia University, in accordance with protocols approved by the Columbia University Institutional Animal Care and Use Committee. Animals were euthanized by CO_2_ asphyxiation with subsequent cervical dislocation to ensure death prior to isolation of BMDMs. For BMDM isolation, the femur and tibia of hindlegs from wildtype mice were resected and crushed using a mortar and pestle. Erythrocytes were lysed using Red Blood Cell Lysis Buffer (Millipore Sigma). Cell suspensions were filtered repeatedly through 70-μm nylon cell strainers (Corning). Bone marrow cells were seeded on untreated plates to allow selection of macrophages. To differentiate monocytes into macrophages, bone-marrow-derived cells were seeded in Dulbecco’s Modified Eagle Medium (DMEM; Gibco) supplemented with 10% fetal bovine serum (FBS; Invitrogen and Atlanta Biologicals), 100 U/ml penicillin, 100 μg/ml streptomycin (both Gibco) and 20% medium previously conditioned for one week with L929 cells. Macrophages were harvested after 6 days of incubation at 37 °C with 5% atmospheric CO_2_. J774 and RAW 264.7 cells were cultured at 37 °C with 5% atmospheric CO_2_ in DMEM supplemented with 10% FBS, 100 U/ml penicillin and 100 μg/ml streptomycin.

### RNA isolation

Total RNA was isolated using TRIzol reagent (Life Technologies) or the RNeasy Plus Mini Kit (QIAGEN) following the manufacturer’s protocol.

### Reverse transcription-quantitative polymerase chain reaction (RT-qPCR)

cDNA was synthesized with SuperScript III Reverse Transcriptase (Invitrogen), essentially following the manufacturer’s instructions and using up to 1 μg total RNA with oligo(dT)_12–18_ primer (Invitrogen). qPCR was then performed with PerfeCTa SYBR Green FastMix (QuantaBio) in accordance with the manufacturer’s instructions, using a Bio-Rad CFX96 or CFX384 Real-Time PCR Detection System (Bio-Rad). Relative cDNA levels were calculated with the ΔΔC_t_ method using *Tubb5* or *Rplp0* as the reference gene^93^. Results were normalized against samples treated only with LPS. To determine the statistical significance of any differences observed between groups, the two-tailed one-sample t-test was used when comparing to reference samples treated only with LPS; the two-tailed unpaired t-test was used for comparisons between other groups. Primer sequences are listed in Supplementary Table ﻿S5, and are largely based on a study by Ramirez-Carrozzi et al*.*^94^.

### Western blot

Cells were seeded at a concentration of 8.0 × 10^5^ cells/ml and treated and/or stimulated as indicated. Cells were washed in phosphate-buffered saline (Gibco) and lysed in Triton lysis buffer (20 mM Tris–HCl, pH 8.0, 150 mM NaCl, 1% Triton-X, 0.5 mM PMSF, 1 mM Na_3_VO_4_). Protein concentrations were quantified with the Micro BCA Protein Assay Kit (Thermo Scientific). Equivalent amounts of total protein were separated by SDS-PAGE and transferred to an Immobilon-P PVDF membrane (Millipore Sigma). Membranes were blocked for 30 min in 5% non-fat dry milk dissolved in Tris-buffered saline with 0.05% Tween 20 (TBS-t; Millipore Sigma), followed by incubation with primary antibody overnight at 4 °C. The following antibodies were used in this study: rabbit anti-phospho-AMPKα (Thr172) (diluted 1:1000; Cell Signaling Technology); rabbit anti-AMPKα (1:1000; Cell Signaling Technology); mouse anti-β-tubulin (1:1000; Millipore Sigma); mouse anti-β-actin (1:5000; Novus Biologicals); rabbit anti-IκB-α (Santa Cruz Biotechnology); and rabbit anti-HIF1-α (1:200; Novus Biologicals). Blots were washed in TBS-t, incubated with horseradish peroxidase-conjugated donkey anti-rabbit IgG or donkey anti-mouse IgG antibody (1:10,000; Cell Signaling Technology) for 30 min, and washed again with TBS-t. Immunoreactive bands were then visualized by chemiluminescence (SuperSignal West Pico Substrate, Thermo Scientific) according to the manufacturer’s instructions.

### Luciferase assays

For HIF1-α luciferase assays, RAW 264.7 cells were seeded at a concentration of 2.0 × 10^5^ cells/ml and transfected with 1 µg pGL2-HRE-Luc (a gift from Navdeep Chandel; Addgene plasmid #26731) using 5 µg PEI Max (Polysciences) per ml^95^. Beginning 24 h after transfection, cells were treated with 5 mM metformin or an equivalent volume of PBS for 6 h prior to stimulation with 100 ng/ml LPS for up to 24 h. Treatments were staggered to permit simultaneous harvest of all samples. Cells were washed in PBS and incubated overnight or longer at -80 °C in 300 μl of Passive Lysis Buffer (Promega). Luciferase activity in each lysate was then assessed using the Dual-Luciferase Reporter Assay System (Promega) as per the manufacturer’s protocol. For NF-κB luciferase assays, RAW 264.7 cells containing the stably integrated pBIIx-Luc NF-κB reporter plasmid were seeded in duplicate at a concentration of 8.0 × 10^5^ cells/ml and pretreated with 1 mM metformin for up to 24 h prior to stimulation with 100 ng/ml LPS for 2 h. Treatments were staggered to permit simultaneous harvest of all samples. Cells were washed in PBS and incubated overnight at -80 °C in 500 μl of Passive Lysis Buffer (Promega). Luciferase activity in each lysate was then assessed using the Dual-Luciferase Reporter Assay System (Promega) as per the manufacturer’s protocol. Results were normalized against untreated samples.

### siRNA knockdown

For knockdown of *Prkaa1*, 1.0 × 10^5^ RAW 264.7 cells were seeded on 24-well plates and transfected with 50 pmol siRNA (Santa Cruz Biotechnology) using 6 µl Lipofectamine 2000 in Opti-MEM (Thermo Fisher Scientific). Medium was removed and cells were incubated directly in transfection mix for 6 h, which was then replaced with fresh growth medium. Twenty-four hours after transfection, cells were pretreated with 5 mM metformin or an equivalent volume of PBS for 6 h prior to stimulation with 100 ng/ml LPS for 2 h. These transfection conditions are based on the knockdown reported by Kim et al*.*^48^.

### RNA sequencing and analysis

Primary BMDMs were seeded in duplicate at a concentration of 8.0 × 10^5^ cells/ml and pretreated with 5 mM metformin for 6 h prior to stimulation with 100 ng/ml LPS for 2 h (Millipore Sigma). RNA was isolated as described above. Quantity and quality of RNA samples were measured by Bioanalyzer (Molecular Pathology core at Herbert Irving Comprehensive Cancer Center, Columbia University). Library construction and sequencing were performed by the JP Sulzberger Columbia Genome Center (Columbia University). Poly-A pull-down was used to enrich mRNAs from total RNA samples and libraries were prepared by using the Illumina TruSeq RNA prep kit. Libraries were then sequenced using an Illumina HiSeq2000 device. All samples were sequenced with 30 million single-end reads. Raw sequencing reads were aligned to the *Mus musculus* genome GRCm38, annotation release 94, using STAR v2.6.1d^96^. SAM files were converted to BAM files and indexed using Samtools v1.9^97^. Quality control was performed using QoRTs v1.3.6 and IGV v2.4.10^98–100^. Aligned reads were counted using the featureCounts setting of the Subread v1.6.3 package^101^. Differentially expressed genes were identified using the DESeq2 v1.22 package in the R environment (v3.4 and higher)^102–104^. Genes were considered differentially expressed when they exhibited a log2-transformed fold-change of at least 2 (LPS vs. untreated) or 0.7 (Metformin + LPS vs. LPS) and an adjusted p-value of less than 0.01. R scripts utilized for statistical analysis and visualization are available from the authors upon request. The RNA-seq data have been deposited in the Gene Expression Omnibus database (accession number GSE131348).

### Ingenuity pathway analysis

Analysis of gene enrichment was performed using IPA (QIAGEN)^73^. For the 1390 genes identified as LPS-responsive, the log2-transformed fold-change, baseMean values and adjusted p-values from the DESeq2 comparison of Metformin + LPS- vs. LPS-treated BMDMs were uploaded to IPA. Enrichment analysis was performed by comparing genes downregulated (log2-transformed fold-change <  − 0.6, padj < 0.01, baseMean > 6.34) by metformin to the background of all 1390 LPS-responsive genes. IPA identified enrichment of metformin-downregulated genes in the gene sets that compose the canonical pathways, biological functions and diseases in Ingenuity’s Knowledge Base library by calculating the ratio of the number of metformin-downregulated genes that mapped to the gene set divided by the total number of considered genes that mapped to the gene set. Fisher’s exact test was then used to calculate the p-value for the enrichment.

## ﻿Supplementary Information

Supplementary Figures.

Supplementary Table S1.

Supplementary Table S2.

Supplementary Table S3.

Supplementary Table S4.

Supplementary Table S5.

## Data Availability

The RNA-seq dataset produced in this study is available at the Gene Expression Omnibus (GEO) repository under accession number GSE131348 (https://www.ncbi.nlm.nih.gov/geo/query/acc.cgi?acc=GSE131348).
