# Supplementary material for: Metformin selectively dampens the acute inflammatory response through an AMPK-dependent mechanism
Source: Sci Rep. 2021 Sep 21;11:18721. doi: 10.1038/s41598-021-97441-x (PMC8455559; doi:10.1038/s41598-021-97441-x)
Supplement: Supplementary file 1 — Supplementary Figures. [file 41598_2021_97441_MOESM1_ESM.pdf]

# Metformin selectively dampens the acute inflammatory response through an AMPK-dependent mechanism

Thomas S. Postler, Vincent Peng, Dev M. Bhatt and Sankar Ghosh

## Supplementary Information

Contents:

- Supplementary Figures S1-S3, with legends
- List of Supplementary Tables S1-S5

Supplementary Fig. S1

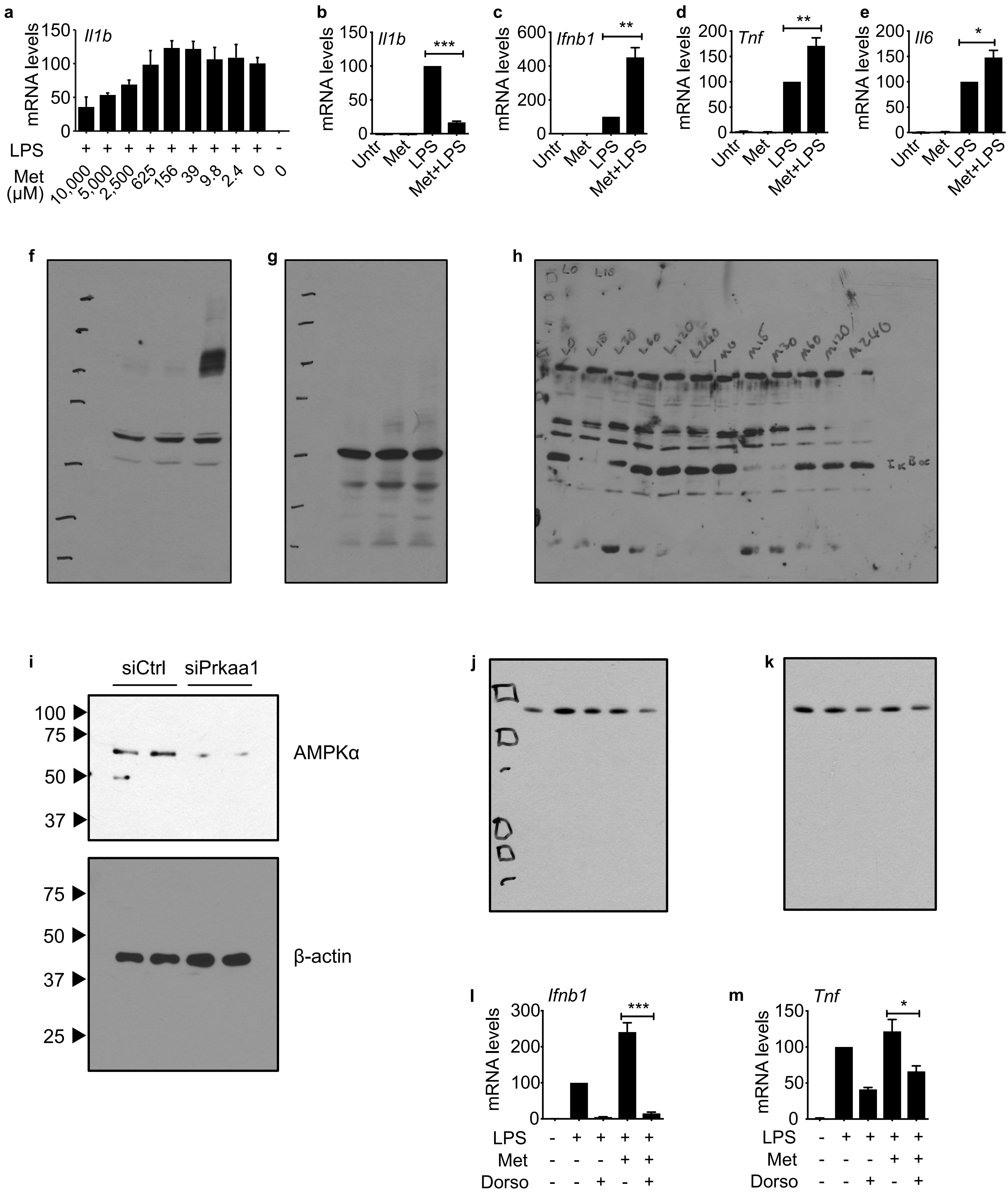

**Supplementary Fig. S1. Metformin alters *Il1b* transcript levels during the acute LPS response.**

**(a)** RT-qPCR with RNA from RAW 264.7 cells, quantifying *Il1b*. Cells were pretreated with the indicated concentrations of metformin for 6 h, followed by stimulation with 100 ng/ml LPS for 2 h. Controls were left untreated. *Tubb5* served as reference gene. Error bars indicate standard deviation of technical triplicates. **(b-e)** RT-qPCR with RNA from J774 cells, quantifying the indicated genes. Cells were pretreated with 5 mM metformin for 6 h, followed by stimulation with 100 ng/ml LPS for 2 h (Met+LPS). Controls were either left untreated (Untr), treated with metformin but not stimulated (Met), or stimulated with 100 ng/ml LPS for 2 h in the absence of metformin pretreatment (LPS). *Tubb5* served as reference gene. Error bars indicate standard error of at least 5 independent experiments. **(f)** Full-length image of the HIF1-α immunoblot shown in Fig. 1f. **(g)** Full-length image of the β-tubulin immunoblot shown in Fig. 1f. **(h)** Full-length image of the IκB-α immunoblot shown in Fig. 1i. **(i)** Western blot of lysates from RAW 264.7 cells transfected with siRNA targeting *Prkaa1* (siPrkaa1) or a control siRNA (siCtrl), probing for total AMPKα and β-actin. Shown are duplicate samples 28 h after transfection. Black arrows indicate molecular weight in kDa. **(j)** Full-length image of phospho-AMPKα (Thr172) immunoblot shown in Fig. 2d. **(k)** Full-length image of total AMPKα immunoblot shown in Fig. 2d. **(l** and **m)** RT-qPCR with RNA from J774 cells, quantifying the indicated genes. Cells were pretreated with 5 mM metformin (Met) and/or 10 μM dorsomorphin (Dorso) for 6 h, followed by stimulation with 100 ng/ml LPS for 2 h. *Tubb5* served as reference gene. Error bars indicate standard error of at least 5 independent experiments. p-values: ***, $p < 0.001$; **, $p < 0.01$; *, $p < 0.05$.

Supplementary Fig. S2

a

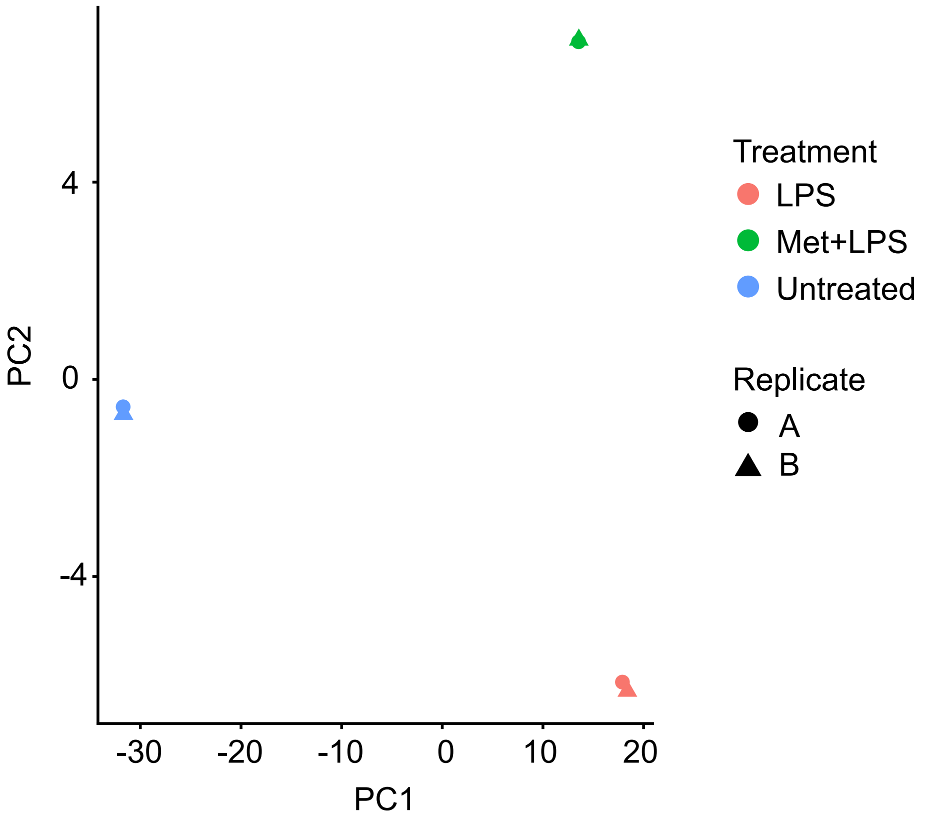


b

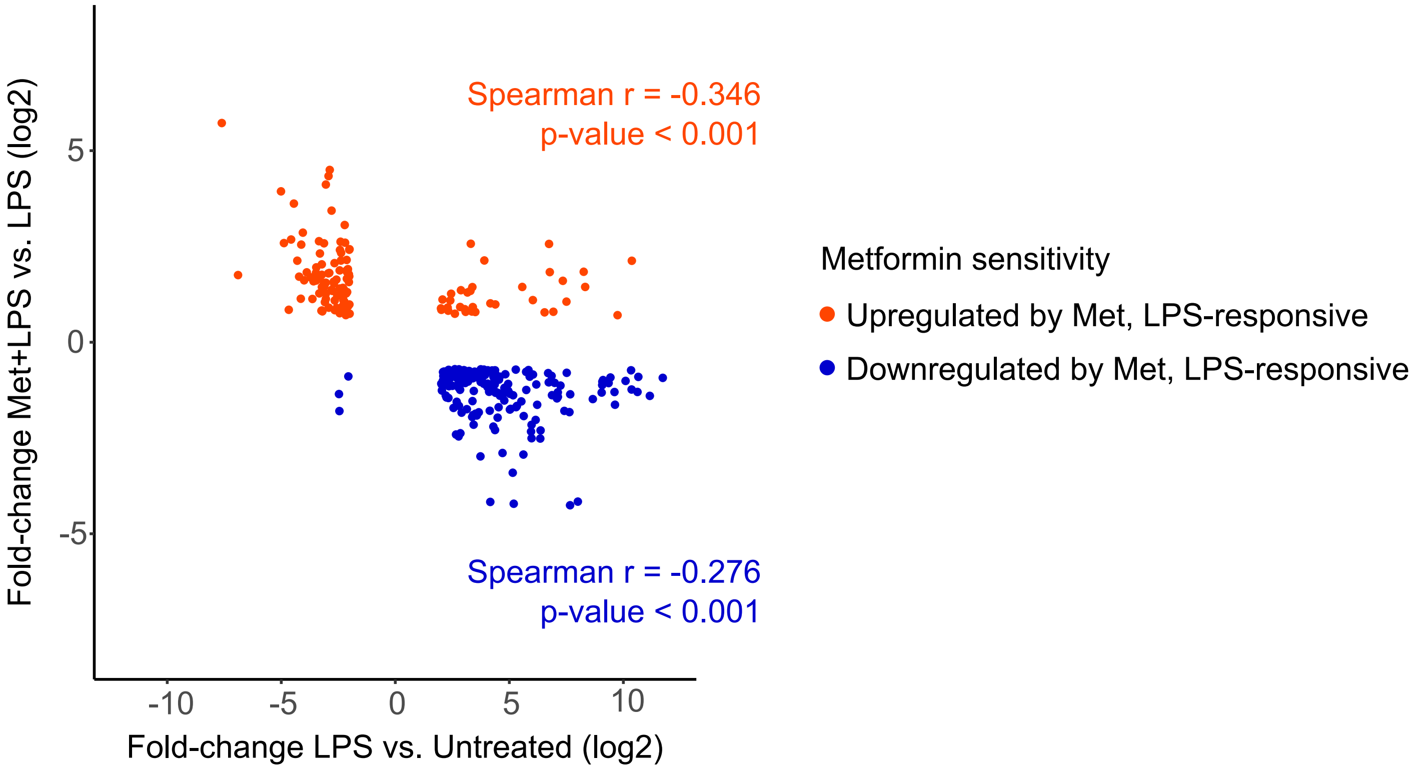

**Supplementary Fig. S2. RNA-seq reveals an unexpectedly broad impact of metformin on the acute LPS response.** Primary BMDMs were pretreated with 5 mM metformin for 6 h, followed by stimulation with 100 ng/ml LPS for 2 h (Met+LPS). Controls were either left untreated (Untreated) or stimulated with 100 ng/ml LPS for 2 h in the absence of metformin pretreatment (LPS). RNA-seq was performed in duplicate for each group. **(a)** Principal Component Analysis of RNA-seq results. **(b)** Scatter plot of log2-transformed fold-changes induced by LPS vs. Untreated cells and fold-changes induced by Met+LPS vs. LPS. Only genes that were both LPS-responsive and metformin-sensitive are shown. Spearman correlation coefficients and corresponding p-values are given separately for genes upregulated by metformin pretreatment (orange) and for genes downregulated by metformin pretreatment (blue).

Supplementary Fig. S3

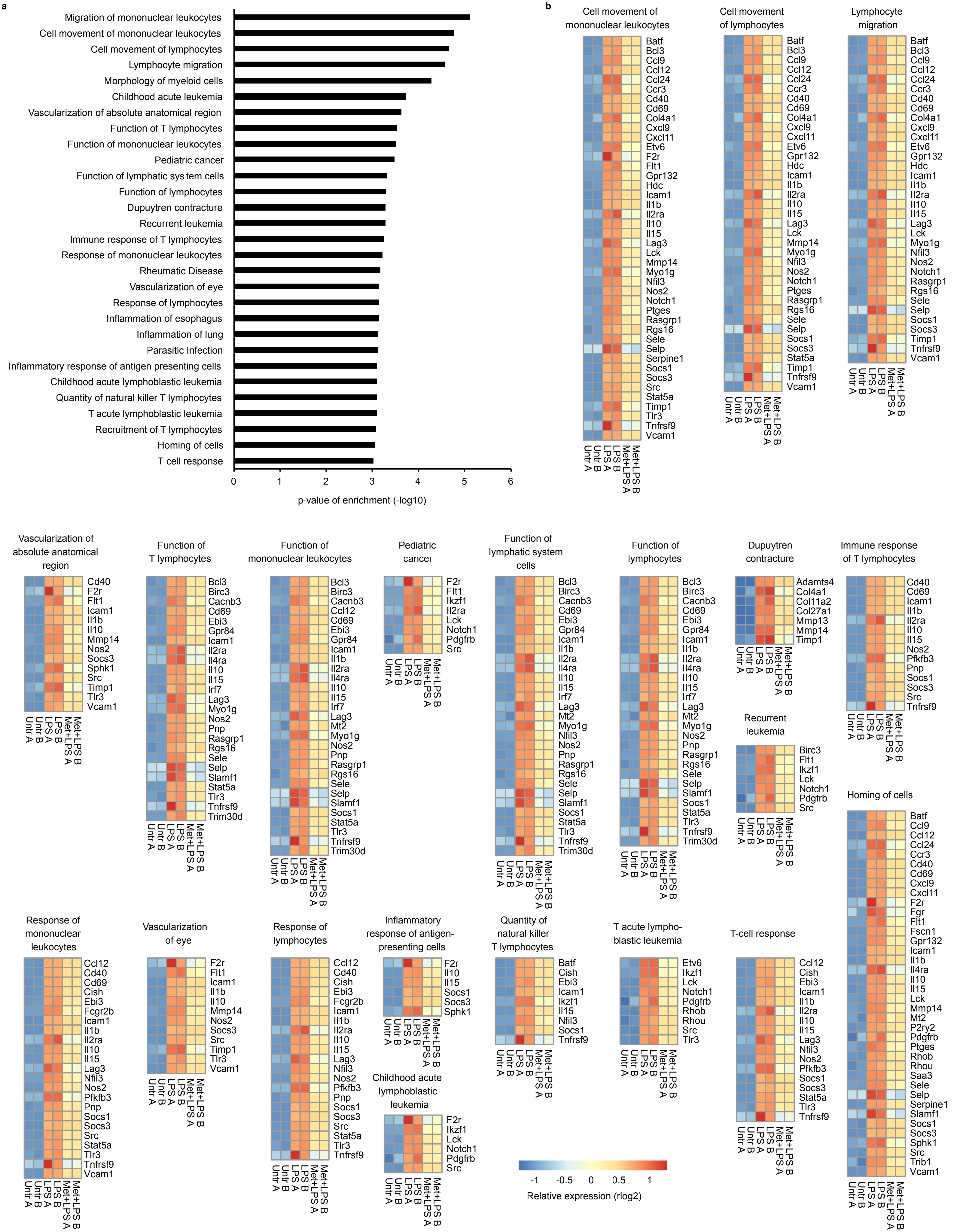

**Supplementary Fig. S3. LPS-responsive genes downregulated by metformin are associated with a wide range of pathways, biological functions and diseases.**

Genes downregulated by metformin were investigated with Ingenuity Pathway Analysis. The set of 1,390 LPS-responsive genes served as background against which enrichment was calculated. **(a)** List of the 29 gene sets enriched for metformin-downregulated genes and associated with biological functions and diseases, with the corresponding p-value of enrichment. **(b)** Alphabetical expression heatmaps of metformin-downregulated genes in the respective gene sets. Colors represent regularized, log2-transformed counts (rlog2) after normalization per row. Shown are the gene sets not presented in Fig. 4.

## LIST OF SUPPLEMENTARY TABLES

**Supplementary Table S1:** DESeq2 results table of LPS vs. Untreated

**Supplementary Table S2:** DESeq2 results table of Met+LPS vs. LPS

**Supplementary Table S3:** DESeq2 results table of Met+LPS vs. LPS for genes that are LPS-responsive and metformin-sensitive

**Supplementary Table S4:** Summary of diseases and functional annotations, IPA analysis

**Supplementary Table S5:** List of qPCR primers used in this study
